# BuMPing Into Neurogenesis: How the Canonical BMP Pathway Regulates Neural Stem Cell Divisions Throughout Space and Time

**DOI:** 10.3389/fnins.2021.819990

**Published:** 2022-01-27

**Authors:** Gwenvael Le Dréau

**Affiliations:** Sorbonne Université, INSERM, CNRS, Institut de la Vision, Paris, France

**Keywords:** bone morphogenetic proteins, SMAD transcription factors, neurogenesis, neural stem cells, self-amplifying divisions, neurogenic divisions

## Abstract

Bone morphogenetic proteins (BMPs) are secreted factors that contribute to many aspects of the formation of the vertebrate central nervous system (CNS), from the initial shaping of the neural primordium to the maturation of the brain and spinal cord. In particular, the canonical (SMAD1/5/8-dependent) BMP pathway appears to play a key role during neurogenesis, its activity dictating neural stem cell fate decisions and thereby regulating the growth and homeostasis of the CNS. In this mini-review, I summarize accumulating evidence demonstrating how the canonical BMP activity promotes the amplification and/or maintenance of neural stem cells at different times and in diverse regions of the vertebrate CNS, and highlight findings suggesting that this function is evolutionarily conserved.

## Introduction

**Figure 1:**
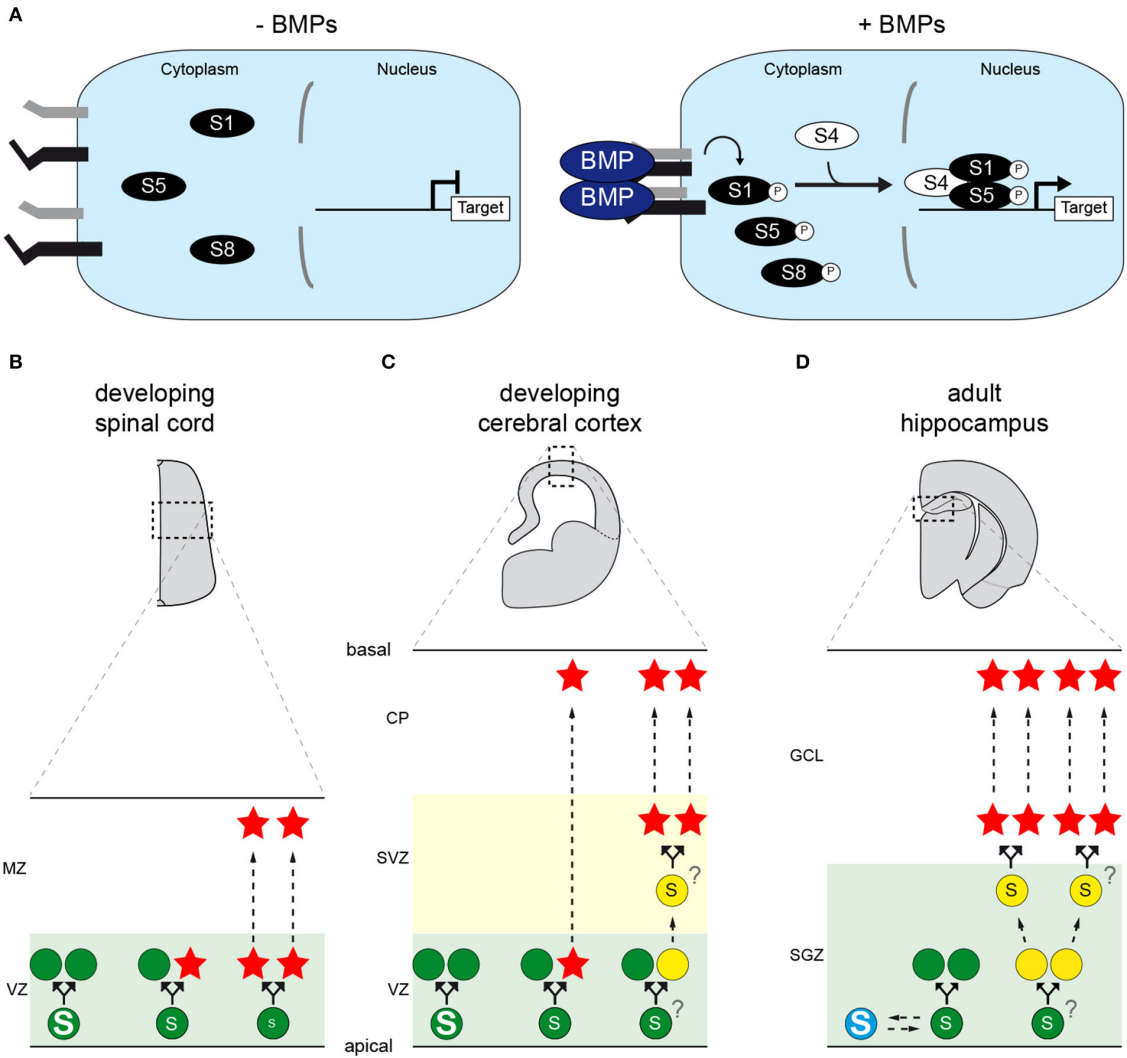
The canonical BMP pathway and its role(s) during vertebrate CNS neurogenesis. **(A)** Representation of the canonical BMP pathway. Dimers of extracellular BMP ligands induce the formation of a tetrameric complex of transmembrane serine/threonine kinase receptors, in which constitutively active type-2 receptors (black) activate type-1 receptors (gray) through phosphorylation. Type-1 receptors in turn phosphorylate serine residues in the carboxy-terminal tail of SMAD transcription factors (SMAD1, SMAD5 or SMAD8/9), enabling them to interact with their co-partner SMAD4. This activated SMAD complex thereby enters the nucleus, recruits co-factors and regulates the transcription of target genes, such as members of the ID family. The duration of exposure to BMPs and their concentration are therefore converted into different levels and/or durations of transcriptional activity. **(B–D)** The canonical BMP activity dictates neural stem cell (NSC) divisions during neurogenesis in the vertebrate CNS. **(B)** During spinal neurogenesis, high levels of SMAD1/5 activity instruct NSCs (green disks) located in the ventricular zone (VZ) to self-amplify, whereas low levels force NSCs to undergo self-consuming divisions, hence depleting their pool and producing neurons (red stars) that will delaminate and migrate toward the mantle zone (MZ). Intermediate levels are proposed to instruct self-renewing neurogenic divisions. **(C)** During cortical neurogenesis, high levels of SMAD1/5 activity stimulate NSC self-amplification, whereas lower SMAD1/5 levels force them to produce neurons that will migrate to the cortical plate (CP). In this region, neurogenesis can occur either directly or indirectly, the latter case giving rise to transit-amplifying basal progenitors (yellow disks) that undergo self-consuming divisions in the subventricular zone (SVZ). Whether the modes of direct and indirect neurogenesis are instructed by comparable or distinct thresholds of SMAD1/5 activity remains unknown. **(D)** In the adult mammalian hippocampus, NSCs located in the sub-granular zone (SGZ) of the dentate gyrus are instructed to remain quiescent (blue disk) in response to canonical BMP signaling. Reducing its activity forces the activation of NSCs, which might self-amplify for several rounds before undergoing self-consuming divisions that produce transit-amplifying progenitors. These in turn produce adult-born neurons that will populate the granule cell layer (GCL). Whether the distinct modes of division that activated NSCs undergo are instructed by comparable or distinct thresholds of SMAD1/5 activity remains unknown.

Bone morphogenetic proteins (BMPs) are a subgroup of secreted molecules belonging to the transforming growth factor β (TGF-β) superfamily (Feng and Derynck, 2005; Schmierer and Hill, 2007). BMPs act as homo- or hetero-dimers to induce the formation of a tetrameric complex of pairs of type-1 and type-2 transmembrane serine/threonine kinase receptors, in which constitutively active type-2 receptors phosphorylate type-1 receptors (Schmierer and Hill, 2007). Once activated, type-1 receptors propagate the signal intracellularly through either a canonical or a non-canonical path (Zhang, 2009; Le Dréau and Martí, 2013). In the so-called canonical BMP pathway, they phosphorylate serine residues in the carboxy-terminal tail of SMAD transcription factors (TFs), enabling them to interact with their co-partner SMAD4 to form a heterotrimeric complex with enhanced nuclear stability (Feng and Derynck, 2005; Schmierer and Hill, 2007). This activated SMAD complex thereby recruits co-factors and modulates the transcription of its target genes (Figure 1A), hence regulating different aspects of cell behavior, in particular cell fate decisions (Feng and Derynck, 2005; Schmierer and Hill, 2007).

In vertebrates, the BMP family consists of numerous ligands (BMP2/4, BMP5/6/7/8, BMP9/10 and the Growth and differentiation factors GDF5/6/7), as is also the case for type-1 receptors (ALK1, ALK2/ACVR1, ALK3/BMPR1A, and ALK6/BMPR1B), type-2 receptors (BMPR2 and ACTR2A/2B, the latter two being shared with the Activin/TGF-β subfamily), and SMADs (SMAD1, SMAD5, and SMAD8/9) (Schmierer and Hill, 2007). These factors are often expressed in overlapping patterns, show some versatility in their molecular interactions and some degree of functional redundancy (Feng and Derynck, 2005; Miyazono et al., 2010).

The canonical BMP pathway plays multiple roles during development of the vertebrate central nervous system (CNS), ranging from the initial specification of the neural tissue to the maturation its cell types (Le Dréau and Martí, 2013; Hart and Karimi-Abdolrezaee, 2020). Recent evidences suggest that BMPs also participate in regulating homeostasis and repair in the adult CNS (Hart and Karimi-Abdolrezaee, 2020). Here, I specifically review their contribution to CNS neurogenesis, focusing mostly on findings obtained *in vivo* in amniote models.

## Neurogenesis, a Matter of Fate

To function, the vertebrate CNS relies on the coordinated activity of billions of neurons, which are produced through a complex process called neurogenesis. Generated from multipotent neural stem cells (NSCs) or from fate-restricted progenitors, new-born neurons exit the cell cycle and progressively differentiate as they delaminate from the germinal zones and migrate basally, plus tangentially in some regions, toward their final location (Götz and Huttner, 2005). This process occurs mainly during embryonic development but is still on-going in discrete regions of the postnatal and adult CNS (Götz and Huttner, 2005; Grandel and Brand, 2013). The production of the appropriate numbers and subtypes of neurons found in the mature vertebrate CNS is regulated both spatially and temporally and is put in balance with the amplification and/or maintenance of the NSC pool (Götz and Huttner, 2005; Obernier and Alvarez-Buylla, 2019; Fischer and Morin, 2021). This equilibrium is achieved *via* a tight control of the distinct modes of division that NSCs and neural progenitors can adopt during neurogenesis.

The neural tissue initially consists of a pseudo-stratified epithelial layer (the ventricular zone, VZ) formed by neuroepithelial cells. These primary NSCs contact both the ventricle and the basal lamina, they undergo mitosis near the apical surface and expand their pool through self-amplifying (symmetric proliferative) divisions that produce two daughter NSCs retaining, as far as we know, the full lineage potential of their mother cell (Götz and Huttner, 2005; Cárdenas and Borrell, 2020). This self-amplification drives the tangential growth of the developing CNS and is the only mode of division at play before neurogenesis.

Developmental neurogenesis is initiated when some NSCs switch from self-amplifying to neurogenic divisions, which come in different flavors. NSCs can undergo self-consuming (symmetric/terminal neurogenic) divisions that directly generate two neurons while depleting the NSC pool, as seen in the developing spinal cord (Saade et al., 2013; Le Dréau et al., 2014). They can otherwise undergo two types of self-renewing divisions that maintain the NSC pool intact by producing a new NSC and another daughter cell with a distinct (asymmetric) identity (Cárdenas and Borrell, 2020). During direct neurogenesis, a NSC produces one neuron *per* division, hence increasing neuron production at a slow pace. During indirect neurogenesis, a NSC instead gives rise to a transient-amplifying progenitor (called basal progenitor, BP) that delaminates from the VZ and divides basally, possibly self-amplifying for several rounds before producing neurons through self-consuming divisions. This mode of indirect neurogenesis is observed in CNS regions undergoing a remarkable radial growth, such as the developing cerebral cortex (Cárdenas and Borrell, 2020). The numbers and subtypes of BPs (intermediate progenitor cells, IPCs; and basal radial glial cells, bRGCs) produced by cortical NSCs (called apical radial glial cells, aRGCs) are strongly correlated to the thickness of the cerebral cortex and to the radial expansion and morphological changes it underwent during evolution (Cárdenas and Borrell, 2020).

In contrast to embryonic NSCs that are permanently cycling, adult NSCs can be found in a reversible state of cell cycle arrest called quiescence. Once activated, adult NSCs appear to divide mostly symmetrically, undergoing either self-amplifying divisions or self-consuming divisions that give rise to transit-amplifying progenitors (called TAPs, TACs or C cells), which in turn produce neurons through self-consuming divisions (Obernier et al., 2018; Obernier and Alvarez-Buylla, 2019).

Therefore, neurogenesis appears to be a matter of fate, whereby the decision of a NSC to undergo self-amplifying, self-consuming, direct or indirect neurogenic divisions has a huge impact on the final neuron output. Throughout the years, many families of intracellular and extracellular actors have been shown to regulate these cell fate decisions during developmental and/or adult neurogenesis (Martynoga et al., 2012; Tiberi et al., 2012; Saade et al., 2018; Obernier and Alvarez-Buylla, 2019; Urbán et al., 2019). One such family corresponds to BMPs.

## BuMPing Into Neurogenesis

Modulating canonical BMP signaling has been reported to alter neuron production in numerous contexts. But in most instances, it is still unclear whether these alterations reflect their involvement in instructing progenitor cell specification at early stages, or if they effectively demonstrate a proper role during neurogenesis *per se*. I thus chose to focus on three tangible examples describing a discrete function of the canonical BMP pathway in regulating NSC fate decisions during neurogenesis: in the spinal cord and cerebral cortex during development, and in the adult hippocampus.

### Promoting Stem Cell Amplification During Spinal Neurogenesis

Emerging from the caudal neural tube, the spinal cord represents the most evolutionarily conserved region of the vertebrate CNS. Before neurogenesis, spinal NSCs are progressively patterned into discrete progenitor domains arrayed along the anterior-posterior (AP) and dorsal-ventral (DV) axes (from dorsal to ventral: roof plate, dorsal progenitor domains dP1-dP6; ventral progenitor domains p0-p2, pMN, p3 and floor plate). Their identity and lineage potential are defined by discrete combinations of patterning homeodomain proteins and proneural basic helix-loop-helix (bHLH) TFs (Le Dréau and Martí, 2012; Sagner and Briscoe, 2019). Secreted from dorsal sources (the roof plate and the surrounding ectoderm), BMPs act as morphogens instructing spinal progenitors to adopt dorsal identities through their canonical pathway (Le Dréau and Martí, 2013; Tozer et al., 2013; Zagorski et al., 2017). Beyond its early role in establishing DV patterning, the canonical BMP pathway also plays a discrete function in regulating neurogenesis later on.

Around the onset of neurogenesis in the developing chick spinal cord, SMAD1/5/8 activity is re-deployed along most of the DV axis, coinciding with the emerging expression of several BMP ligands, in particular BMP7, in intermediate and ventral progenitor domains (Le Dréau et al., 2012). High, intermediate and low levels of nuclear SMAD1/5 activity correlate, respectively, to self-amplifying, direct self-renewing and self-consuming divisions (Figure 1B). Gain- and loss-of-functions experiments revealed that SMAD1/5 activity promotes self-amplifying divisions while restraining neurogenic ones. In particular, inhibiting SMAD1/5 activity first causes a premature increase in neurogenic divisions (especially self-consuming ones) at the expense of self-amplifying ones, subsequently depleting the progenitor pool and ultimately reducing the global production of spinal neurons (Le Dréau et al., 2014). In agreement with these findings, reducing the expression of either BMP7, SMAD1, SMAD5 or overexpressing the inhibitory SMAD6 and SMAD7 impairs the generation of spinal neurons in chick (Hazen et al., 2011; Le Dréau et al., 2012, 2014, 2018). The canonical BMP activity is thus crucial to properly balance progenitor expansion and neuron production during chick spinal neurogenesis. Similar phenotypes are observed in mutant mice lacking either BMP7, SMAD1 or SMAD5 (Hazen et al., 2012; Le Dréau et al., 2012), suggesting that this role is evolutionarily conserved in amniotes.

However, this stem cell-promoting role appears to be context-dependent. Indeed, the generation of spinal neurons deriving from progenitors expressing either the proneural bHLH TF ATOH1 (dP1) or high levels of ASCL1 (dP3, dP5, and p2) is highly sensitive to variations in canonical BMP activity. Conversely, the production of spinal neurons deriving from progenitors expressing PTF1a (dP4), NEUROG1 (dP2, dP6-p1), NEUROG2 (pMN) and possibly NEUROG3 (p3) is much less affected by such variations (Hazen et al., 2011, 2012; Le Dréau et al., 2012, 2018; Andrews et al., 2017). A mechanistic explanation for this context-dependent requirement was recently proposed (Le Dréau et al., 2018). Once activated, SMAD1/5 positively regulate the expression of ID2 (Inhibitor of DNA-binding 2), and possibly of other ID members. IDs physically sequester the bHLH E proteins TCF3 and TCF12, hence blocking their ability to dimerize with the proneural TFs. Interestingly, E proteins share the preferential binding of ATOH1 and ASCL1 for *CAGSTG* DNA motifs (Lin et al., 2010; Castro et al., 2011; Klisch et al., 2011; Lai et al., 2011; Borromeo et al., 2014; Pfurr et al., 2017). By restraining the availability of E proteins, high SMAD1/5 activity thus indirectly impedes ASCL1/ATOH1 from triggering neurogenic divisions, hence promoting self-amplifying divisions. When SMAD1/5 activity is instead reduced, E proteins are released and heterodimerize with ASCL1/ATOH1, forcing spinal progenitors to undergo neurogenic divisions (Le Dréau et al., 2018). Inversely, E proteins show lower affinity for the *CADATG* motifs preferentially bound by NEUROG1/2/3 (Seo et al., 2007; Madelaine and Blader, 2011; Borromeo et al., 2014). The ability of NEUROG TFs to trigger neurogenic divisions appears to be less dependent on, and even somewhat restrained by, E proteins, making them less sensitive to variations in canonical BMP signaling (Le Dréau et al., 2018). Therefore, the molecular machinery instructing the identity and lineage potential of spinal progenitors dictates their specific requirement on canonical BMP signaling to pace neurogenesis.

### Promoting Stem Cell Amplification During Cortical Neurogenesis

Emerging from the pallium, which itself represents the dorsal part of the telencephalon, the cerebral cortex represents the most evolutionarily divergent region of the mammalian CNS, with comparable structures being only found in the amniote clade (Goffinet, 2017; Briscoe and Ragsdale, 2018; García-Moreno and Molnár, 2020). During early mammalian brain development, various BMP ligands (BMP2/4/5/6/7) are expressed and secreted by discrete regions of the dorsal telencephalon, the cortical hem and the choroid plaque, from where they act as morphogens to pattern the dorsal telencephalic midline (Furuta et al., 1997; Grove et al., 1998; Hebert et al., 2002; Hébert and Fishell, 2008). Accordingly, most of these ligands are detected in the developing cerebrospinal fluid known to nurture the survival and proliferation of cortical aRGCs (Lehtinen et al., 2011).

Early *in vitro* studies performed on explants and dissociated cortical progenitors suggested that BMPs inhibit their proliferation and stimulate neuronal differentiation (Li et al., 1998; Mabie et al., 1999). The phenotypes of brain over-proliferation and premature differentiation, respectively, reported for transgenic mice expressing constitutively active forms of ALK3 or ALK6, suggested a more complex contribution (Panchision et al., 2001). In most instances the phenotypes obtained after deleting one BMP family member were poorly informative, causing either early embryonic lethality (BMP2/4, SMAD1/5) (Winnier et al., 1995; Zhang and Bradley, 1996; Yang et al., 1999; Tremblay et al., 2001), no obvious brain defects (BMP5/6/9, GDF5/6 or FoxG1^Cre^; BMP4^fl/fl^) (Kingsley et al., 1992; Storm et al., 1994; Solloway et al., 1998; Hebert et al., 2003; Settle et al., 2003; Ricard et al., 2012), or only mild ones limited to the dorsal midline (GDF7, SMAD8/9, FOXG1^Cre^;ALK3^fl/fl^) (Lee et al., 1998; Hebert et al., 2002; Hester et al., 2005).

The first conclusive evidence of the physiological implication of BMP signaling in cortical neurogenesis came from a study revealing that BMP7 null mice are microcephalic (Segklia et al., 2012). The cortices of BMP7 null embryos show a normal organization and layering but are thinner and contain lower numbers of neurons. At mid-corticogenesis, the amount of cortical aRGCs is also reduced, as are their abilities to proliferate and to sustain neurosphere formation *in vitro* (Segklia et al., 2012). Remarkably, reducing the expression of both SMAD1 and SMAD5 in mouse neural progenitors, using a *Nestin:Cre* driver triggering recombination at early stages, also causes microcephaly (Najas et al., 2020). These SmadNes mice moreover show an increased production of early-born cortical projection neurons at the expense of late-born ones, which correlates with the premature differentiation and depletion of the pools of cortical progenitors, including both aRGCs and IPCs (Najas et al., 2020). This phenotype, combined with the detection of higher SMAD1/5 activity in mitotic aRGCs than in IPCs, suggested that these two canonical BMP effectors stimulate stem cell maintenance during mammalian corticogenesis.

This idea was further tested in the chick hyperpallium. Whether this dorsal pallial derivative specific of birds should be considered homologous to the mammalian neocortex remains debated (Goffinet, 2017; Briscoe and Ragsdale, 2018; García-Moreno and Molnár, 2020). Nevertheless, neurogenesis in the chick hyperpallium involves progenitor cell types, cellular events and a temporal sequence similar to those described in mammals (Cárdenas and Borrell, 2020). In the developing chick hyperpallium, higher levels of SMAD1/5 activity correlate with aRGC self-amplifying potential (Najas et al., 2020). Inhibiting their expression during early neurogenesis reduces self-amplifying divisions in favor of neurogenic ones, resulting in premature neuronal differentiation as observed in mouse. Conversely, enhancing SMAD1/5 activity stimulates aRGC self-amplifying divisions and restrains their neuronal commitment (Figure 1C). Mechanistically, SMAD1/5 appear to stimulate aRGC amplification during both chick and mouse corticogenesis by positively regulating and recruiting YAP (Najas et al., 2020), a key transcriptional co-factor of the Hippo pathway known to regulate organ size (Yu et al., 2015). Thus, the canonical BMP pathway also promotes stem cell amplification during corticogenesis, and this function is likely conserved throughout the amniote lineage, at least between birds and mammals.

### Sustaining Stem Cell Quiescence During Adult Neurogenesis

The ability of the brain to produce new neurons during adult life is apparently conserved throughout the vertebrate lineage, being more widespread in amphibians and fish than in amniotes (Grandel and Brand, 2013). In the mammalian brain, adult NSCs are found in two neurogenic niches: the subgranular zone (SGZ) of the hippocampal dentate gyrus and the ventricular-subventricular zone (V-SVZ) of the lateral telencephalic ventricle walls (Obernier and Alvarez-Buylla, 2019; Urbán et al., 2019). Various BMPs (BMP2/4/5/6/7) and extracellular antagonists (such as NOGGIN and CHORDIN) are found in these two adult neurogenic niches, being expressed by adult NSCs themselves or secreted by their microenvironment, including the choroid plexus, ependymal cells and blood vessels (Urbán and Guillemot, 2014; Obernier and Alvarez-Buylla, 2019). Adult NSCs express various BMP receptors and show nuclear SMAD1/5/8 activity, which demonstrates their responsiveness to BMPs.

The ability of BMPs to regulate adult neurogenesis has been well-characterized in the SGZ of the dentate gyrus, where hippocampal NSCs reside (Urbán et al., 2019). There, inhibiting BMP signaling using intracerebral injections of NOGGIN or through the selective deletion of ALK3 or SMAD4, transiently enhances NSC self-amplification but subsequently causes their depletion, thereby impairing the long-term production of newborn granule neurons (Bonaguidi et al., 2008; Mira et al., 2010). Therefore, BMP signaling actively supports hippocampal NSC quiescence. Apparently, its activity can stimulate a return to quiescence at multiple stages of this neurogenic sequence (Bond et al., 2014).

Mechanistically, maintaining adult hippocampal NSCs in quiescence requires the continuous degradation of ASCL1 protein levels (Urbán et al., 2016). This process is triggered when its dimerizing co-factor E47 is sequestered by IDs, at least ID4 (Blomfield et al., 2019). In agreement with the fact that IDs are direct transcriptional targets of SMAD1/5 in numerous contexts, treatment of hippocampal NSCs *in vitro* with BMP4 increases the transcript levels of all four ID members, triggering ASCL1 protein degradation (Blomfield et al., 2019). Altogether, these findings support the notion that BMPs promote adult NSC quiescence through their canonical pathway (Figure 1D).

This function might be conserved throughout the vertebrate lineage. Indeed, in the adult zebrafish telencephalon, ID1 is mostly expressed by quiescent NSCs and its overexpression stimulates NSC quiescence *in vivo*, while its knockdown increases neurogenesis (Rodriguez Viales et al., 2015). The expression of ID1 in these adult zebrafish NSCs depends on an evolutionarily conserved cis-regulatory DNA motif that is controlled by BMPs in a SMAD-dependent manner (Zhang et al., 2020).

## Conclusions

There is accumulating evidence that the canonical BMP pathway is a master regulator of neurogenesis in vertebrates, orchestrating this process in the CNS throughout space, time and possibly throughout evolution. As highlighted above, this pathway stimulates the expansion of the NSC pool in both the cerebral cortex and spinal cord during amniote development. In the adult mammalian hippocampus and zebrafish telencephalon, it instead limits the numbers of NSCs by promoting their quiescence. One might thus reason that the canonical BMP pathway plays different roles in the adult CNS or during its development. It might however be argued that this pathway has the same function in all these contexts, considering that its activity always restrains NSCs from progressing into the neurogenic lineage.

Whether the canonical BMP pathway plays the same role in other neurogenic contexts remains subject to debate. For instance, BMP signaling was initially proposed to promote stem cell maintenance in the adult V-SVZ (Lim et al., 2000), but later studies led to divergent conclusions (Bonaguidi et al., 2008; Colak et al., 2008; Silva-Vargas et al., 2016). There are also a few regions of the developing CNS in which BMPs appear to restrain or instead stimulate neurogenesis in a more complex, stage-dependent manner (Alder et al., 1999; Angley et al., 2003; Rios et al., 2004; Krizhanovsky and Ben-Arie, 2006; Jovanovic et al., 2018). These context-dependent effects appear to depend on the identity of the molecular actors involved, both at the level of the BMP pathway and downstream. For instance, the type-1 BMP receptors ALK2/3/6 often show complementary expression patterns and non-redundant effects on neurogenesis, both during development and in adult neurogenic niches (Panchision et al., 2001; Caronia et al., 2010; Mira et al., 2010; Choe et al., 2013). This could imply that the distinct BMP ligands and receptors differentially regulate SMAD1/5/8 activity. On the other hand, NSCs and neural progenitors seem to differentially respond to SMAD1/5/8 activity on the basis of intrinsic molecular differences, such as the identity of the proneural bHLH TFs that they express (Le Dréau et al., 2018).

Therefore, fully understanding the role(s) played by the canonical BMP pathway during neurogenesis will require addressing the discrete contribution of each ligand and downstream effector. Given the diversity of members existing at all levels of the BMP signaling cascade, this represents a laborious task. Such hard work might however offer the possibility to harness the ability of the canonical BMP pathway to regulate neurogenesis for regenerative medicine.

## Author Contributions

The author confirms being the sole contributor of this work and has approved it for publication.

## Funding

This work was supported by Grants from IHU FOReSIGHT (ANR-18-IAHU-01) and LabEx LIFESENSES (ANR-10-LABX-65).

## Conflict of Interest

The author declares that the research was conducted in the absence of any commercial or financial relationships that could be construed as a potential conflict of interest.

## Publisher's Note

All claims expressed in this article are solely those of the authors and do not necessarily represent those of their affiliated organizations, or those of the publisher, the editors and the reviewers. Any product that may be evaluated in this article, or claim that may be made by its manufacturer, is not guaranteed or endorsed by the publisher.
